# Clever Cattle Parasite Captures Cell Division Machinery

**DOI:** 10.1371/journal.pbio.1000498

**Published:** 2010-09-28

**Authors:** Mason Inman

**Affiliations:** Freelance Science Writer, Karachi, Pakistan

**Figure pbio-1000498-g001:**
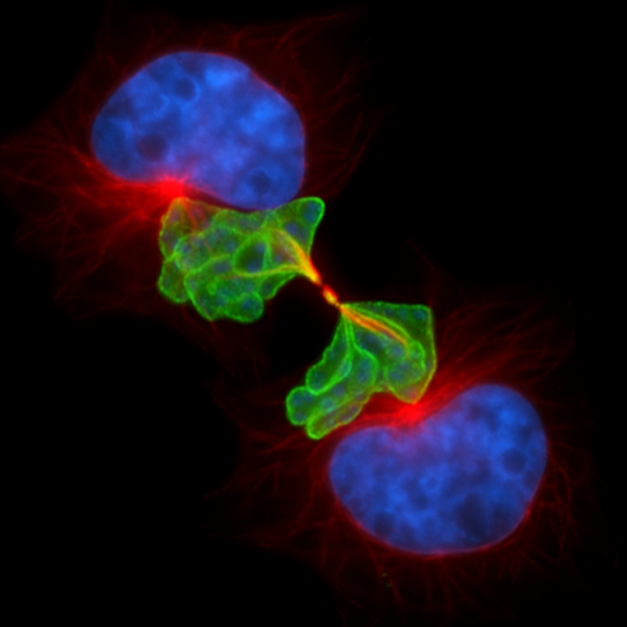
Perfectly positioned for division: the intracellular parasite *Theileria* induces uncontrolled proliferation of its host cell, but how does it ensure it is divided along with the daughter cells at each host cell division?

[Fig pbio-1000498-g001]Parasites have developed clever and diverse ways of making sure they're carried from one generation to the next and spread through their hosts' bodies—not surprising since their lives depend on it. When some viruses want to ensure safe passage to the next generation, they incorporate their DNA right into the host's genome. Others link their genome to host chromosomes so it gets carried along whenever the host's cells divide. The protozoan parasite *Toxoplasma gondii*, on the other hand, rides along in its host's immune cells, allowing it to spread farther and faster through the body.

But there's one especially clever parasite—*Theileria*, a genus of protozoa that infects cattle across the world—that's known to cause a cancer-like disease, making its host's white blood cells divide rapidly, as its way of quickly passing itself on to many of its host's cells and increasing its chances of continuing its life cycle.

*Theileria*'s life cycle, like that of many parasites, is convoluted. *Theileria* first enters cattle when they're bitten by ticks infected with the parasite. In cattle, it colonizes white blood cells, and then passes into red blood cells. That puts it in the right place to get passed on to ticks when they feed on infected cattle. Once a tick takes a blood meal, *Theileria* moves first into the insect's gut, and then on to their salivary glands, from which it can be transmitted to other cattle while the tick is feeding and start its cycle anew.

It is during the key stage of the *Theileria* life cycle when it lives inside the cow's white blood cells that it forces host cells to divide over and over. During this stage, it takes on a form known as a schizont, a super-cell with many replicating nuclei inside. When the cattle's cells divide, the schizont also divides in two, with each half passed on to the host's daughter cells.

If the parasite wound up in only one daughter cell after each host cell division, it would never increase the number of blood cells it colonized and thus would have a small chance of getting ingested by feeding ticks and being passed on from there. But that's not what happens. *Theileria* manages to insert itself into both daughter cells in each cell division, exponentially multiplying its numbers in the process. But just how it manages this impressive feat has remained unclear.

A new study by Conrad von Schubert and colleagues in the group of Dirk Dobbelaere shows that *Theileria* inserts itself into both daughter cells by co-opting parts of the host cell's division machinery. Through this mechanism, the parasite gets treated much like the host's own chromosomes, so that the parasitic super-cell gets neatly divided in two for delivery to each daughter cell.

During the host cell's normal cell division cycle, known as mitosis, long strands of DNA molecules condense into chromosomes visible under a microscope. The chromosomes then line up near the center of the cell before being divided into duplicate halves. Each half is then dragged to opposite sides of the cell so when the cell divides, each daughter cell has a full complement of chromosomes. Protein complexes called microtubules—long, tentacle-like structures—play a key role in this process, grabbing hold of the chromosomes, and pulling them toward the opposite sides of the cell before the cell splits in two. Acting almost like a chromosome in disguise, the *Theileria* parasite uses the same microtubules to be pulled towards the opposing ends of the dividing cell. But how does it deal with the next step: cell division? A key molecule involved in orchestrating this dance, called Polo-like kinase 1 (Plk1), helps organize the microtubules and determine where the cell pinches together to split in two. New chemical tools, which quickly and completely inactivate Plk1 at specific time points during cell division, allowed Conrad von Schubert and colleagues to test how *Theileria* might interact with this enzyme.

To investigate Plk1's contribution to this process, von Schubert and colleagues used fluorescent antibodies to label Plk1, microtubules, and a molecule on the outside of *Theileria* cells known as *Theileria annulata* surface protein 1 (TaSP1). To see where the parasite and key parts of the host's cell division machinery were at various stages of cell division, they stopped the process at particular points. During a preparatory stage of cell division called G2, Plk1 is abundant in host cells. These authors found that at this point *Theileria* scavenges Plk1 and attaches it to its surface. As the host cell starts mitosis, *Theileria* releases Plk1, but only transiently. In the next stage of division known as anaphase—at which point the host cell's chromosomes divide into two equal halves and each half moves into one side of the cell—*Theileria* again rapidly dons Plk1 on its surface. This seems to ensure that a special set of host microtubules, assembling between the separated chromosomes in preparation for cell division, become tightly associated with the schizont. The cell then pinches together at exactly this place and, at the end of the process, as the thin remaining bridge keeping the daughter cells together is severed, the schizont is also cleaved. In this way, *Theileria* appears to ensure that it also gets split in two and passed on to both of the new host cells.

It seems that by cloaking itself with Plk1, *Theileria* is, in a sense, disguising itself as part of the host cell's division machinery. It may also be triggering the cell to divide right at the spot where the parasite sits, the authors argue.

In all, the study reveals how this unique parasite has found evolutionary success through a different route than many other parasites. Rather than attaching itself directly to the host's chromosomes, it manipulates key parts of the host's cell division machinery. This helps ensure that when the cells divide, the parasite will be poised and ready to hitch a ride.

**von Schubert C, Xue G, Schmuckli-Maurer J, Woods KL, Nigg EA (2010) The Transforming Parasite** ***Theileria*** **Co-Opts Host Cell Mitotic and Central Spindles to Persist in Continuously Dividing Cells. doi: 10.1371/journal.pbio.1000499**

